# Nitrogen Recycling and Flowering Time in Perennial Bioenergy Crops

**DOI:** 10.3389/fpls.2013.00076

**Published:** 2013-04-22

**Authors:** Christopher Schwartz, Richard Amasino

**Affiliations:** ^1^Great Lakes Bioenergy Research Center, University of Wisconsin-MadisonMadison, WI, USA

**Keywords:** nitrogen recycling, perennialism, switchgrass, flowering time, dormancy, bioenergy crops

## Abstract

Perennials have a number of traits important for profitability and sustainability of a biofuel crop. Perennialism is generally defined as the ability to grow and reproduce in multiple years. In temperate climates, many perennial plants enter dormancy during winter and recycle nutrients, such as nitrogen, to below ground structures for the next growing season. Nitrogen is expensive to produce and application of nitrogen increases the potent greenhouse gas NO*_x_*. Perennial bioenergy crops have been evaluated for biomass yields with nitrogen fertilization, location, year, and genotype as variables. Flowering time and dormancy are closely related to the N recycling program. Substantial variation for flowering time and dormancy has been identified in the switchgrass (*Panicum virgatum* L.) species, which provides a source to identify the genetic components of N recycling, and for use in breeding programs. Some studies have addressed recycling specifically, but flowering time and developmental differences were largely ignored, complicating interpretation of the results. Future studies on recycling need to appreciate plant developmental stage to allow comparison between experiments. A perennial/annual model(s) and more environmentally controlled experiments would be useful to determine the genetic components of nitrogen recycling. Increasing biomass yield per unit of nitrogen by maximizing recycling might mean the difference for profitability of a biofuel crop and has the added benefit of minimizing negative environmental effects from agriculture.

## Perennialism

Providing sufficient biomass to replace a significant portion of fossil fuel use is a major challenge for the bioenergy industry. Bioenergy crops need to be profitable for the grower and environmentally sustainable, while not competing with food crops. Therefore, low productivity land with marginal soils have been targeted as a primary location for growing bioenergy crops.

Certain traits of perennial plants can contribute to the sustainability of a bioenergy industry. Perennials are generally defined as plants that live for many years and reproduce in multiple years (iteroparity), compared to annuals that reproduce once and then die (semelparity). Perennials retain shoots that do not flower at the end of the season, and instead develop and flower the following season (in grasses and other herbaceous temperate perennials these shoots are at the crown of the plant or in underground stems). Perenniality is likely ancestral to the annual growth habit, since flowering plants originated in warmer eras, which permit continuous growth. Dormancy and the annual growth habit are two adaptations that plants have evolved to survive as they adapted to cooler climates over geologic time.

Perennials have certain advantages over annuals as bioenergy crops. Perennials do not require the energy inputs for planting every season. Growing perennials greatly reduces the capacity for erosion and in fact typically increases soil carbon. The increased soil carbon is due to the deep and extensive root systems of certain perennials; such root systems also permit growth in drier regions. Perennials typically require less fertilizer than annual crops. The reduced requirement for fertilizer is particularly apparent in perennials that have evolved a yearly nutrient recycling and shoot “die-back” program as an adaptation to growth in temperate climates. The recycling saves nutrients such as N and P for next seasons growth and results in senescing shoots with lower N and P content, which facilitates biomass processing. One Miscanthus plot has been harvested continually for 14 years with no inputs and no decrease in yield (Christian et al., 2008).

## Nutrient Recycling in Perennial Bioenergy Crops

In preparation for the following season, perennials cease vegetative growth and initiate flowering mid season. In environments that require a period of dormancy during the year (drought/winter), perennials recycle a portion of their nutrients to below ground structures for growth once the dormant period has passed (McKendrick et al., 1975; Clark, 1977; Hayes, 1985; Beale and Long, 1997; Lemus et al., 2008). How recycling is initiated and the factors regulating recycling remain unknown, but optimizing this trait could result in a significant increase in yields while at the same time reducing inputs. Furthermore, nutrient recycling and storage allows for perennial species to initiate growth immediately in the spring outcompeting annuals that need to emerge from seeds and send out roots to acquire nutrients.

One way to increase perennial biomass production is to extend the vegetative phase by delaying flowering and dormancy, but this may also have a negative effect on end of the season recycling. Harvest date has a large effect on biomass quality and stand longevity; later harvest dates increase quality and longevity by allowing the recycling program to be completed (Sanderson et al., 1999; Reynolds et al., 2000; Muir et al., 2001; Mulkey et al., 2006). In practice, it is best to harvest after a killing frost, since any recycling would cease at that point. When biomass was harvested green, N content exceeded 1.5% compared to less than 0.5% if harvested in the winter, and delaying harvest until at least late summer is advantageous for long-term sustainable biomass production (Casler and Boe, 2003; Adler et al., 2006; Heaton et al., 2009).

In most environments, nitrogen and precipitation are the limiting factors for plant growth, and the primary energy input for crops is usually nitrogen fertilizer (Biermann et al., 1999; Monti and Venturi, 2003; Boehmel et al., 2008). Nitrogen is energy intensive to produce and requires energy for application. Applied agricultural nitrogen is also a primary source of NO*_x_* greenhouse gases. One study estimated that the NO*_x_* produced from fertilizing bioenergy crops would mitigate any potential carbon dioxide decrease, since NO*_x_* species are more potent greenhouse gases than CO_2_ (Crutzen et al., 2008).

With sufficient precipitation, *Miscanthus giganteus* out produces most other bioenergy crops (Heaton et al., 2009), but the clonal nature and lack of natural variation could be detrimental when challenged with biotic stresses or drought. Native warm-season prairie grasses, such as switchgrass and big bluestem, are two species being developed for sustainable biomass production. These species are native to large regions of North America, and are adapted to the regions where dedicated bioenergy will be grown, and thus there are substantial genetic variations for breeding programs.

Several studies have demonstrated movement of nitrogen from shoots to below ground structures in the later part of the growing season in both switchgrass and big bluestem, sometimes over 50% (Hayes, 1985; Tufekcioglu et al., 2003; Lemus et al., 2008; Yang et al., 2009; Garten et al., 2010). Thus these species have a valuable trait for a dedicated bioenergy crop-robust end of the season N recycling. Additionally, switchgrass, big bluestem, and several other C4 warm-season perennial grasses can recycle of up to 30% of shoot nitrogen during drought, presumably as a protective measure for plant survival (Hayes, 1985; Heckathorn and DeLucia, 1994, 1996). Determining the signals leading to drought induced recycling would enable a comparison to end-of-season recycling.

A number of publications address N application and yield, and a subset are listed in Table 1. Variables in most of the studies include years, N application rate, harvest regime, location, and/or cultivar. While the specific techniques differ, nearly every study observed a decrease in total N in above ground tissue during the second half of the growing season, and in some cases it was directly demonstrated that below ground N content increased (Lemus et al., 2008). At ground level or below ground biomass can be 84% of total plant biomass and consists of the crown, rhizomes (underground stems), and roots, providing a large sink for N storage (Frank et al., 2004). In two studies, it was shown that half of the aboveground N was translocated to rhizomes and roots by the time the plants became dormant (Garten et al., 2010; Kering et al., 2012).

**Table 1:** **Studies investigating N dynamics and yield in switchgrass**.

Variables						
N recycling	Author year	Location	Genotype	N treatment	Harvest	Years
Y	Yang et al. (2009)	N	Y (31)	N	Y	N
Y	Garten et al. (2010)	N	Y (4)	N	N	N
Y	Kering et al. (2012)	N	N (Alamo*)	Y	N	Y
Y	Lemus et al. (2008)	Y	N (CIR)	**Y**	Y	Y
Y	Heaton et al. (2009)	Y	N (CIR)	N	Y	Y
Y	Staley et al. (1991)	Y	N	Y	Y	Y
Y	Stout and Jung (1995)	Y	N (CIR)	**Y**	Y	Y
Y	Guretzky et al. (2011)	Y	N (Alamo, LL)	**Y**	Y	Y
N	Muir et al. (2001)	Y	N (Alamo, LL)	**Y**	N	Y
N	Vogel et al. (2002)	Y	Y (CIR)	**Y**	Y	Y
N	Ma et al. (2001)	Y	N (Alamo, LL)	**Y**	Y	Y
N	Reynolds et al. (2000)	N	Y (6)	N	Y	Y
N	Fike et al. (2006)	Y	Y (2 UP, 2 LL)	**Y**	Y	Y
N	Thomason et al. (2005)	Y	N	**Y**	Y	Y
N	Boehmel et al. (2008)	N	N (Kanlow)	**Y**	N	Y
N	Lee et al. (2007)	N	N	**Y**	Y	Y
N	Mulkey et al. (2006)	Y	N	Y	Y	Y
N	Sanderson et al. (1999)	Y	Y	N	Y	Y

*Bold indicates a significant effect on yield with N application*.

* A selection derived from the cultivar Alamo.

From an agronomic point of view, robust recycling will allow for a lower rate of N application, while generating good yields of biomass. Many studies show a positive correlation of increased biomass with increasing N application (Table 1, Bold). These studies also establish that higher rates of N application lead to increases of N in harvested tissue and a decrease in the amount of biomass produced per gram of N applied (Staley et al., 1991; Muir et al., 2001; Vogel et al., 2002; Lewandowski et al., 2003; Mulkey et al., 2006; Lemus et al., 2008; Guretzky et al., 2011). For example, in Guretzky et al. (2011), application of 225 kg Nha-1 increased switchgrass biomass by 85%, yet N content of harvested biomass increased by 182%, compared to no N applied. Thus robust N recycling will produce biomass with less N, mitigating some of the environmental damage caused by NO*_x_* resulting from N application.

One complication of interpreting N use studies is the age and developmental stage of the plants, because results will be affected by both plant size and developmental stage and the impact of developmental stages on physiological/biochemical analyses can be larger than genetic differences in herbaceous annual crops. Using a developmental index, such as that developed by Moore et al. (1991), allows for comparison across genotypes and studies. Recently, a standardization protocol for switchgrass sample collection has also been developed (Hardin et al., 2013). Determining the maximum and minimum N content in the plant is key to determining resorption efficiency. The maximum value is likely to be at the initiation of reproductive structures, presumably before whole plant senescence begins. In addition, there are overall mass decreases at the end of the season, due to carbohydrate depletion and translocation, thus corrections need to be made to get an accurate N recycling estimate (Van Heerwaarden et al., 2003; Heaton et al., 2009).

Many studies have also evaluated natural genetic variation for N recycling in switchgrass. However, large environmental effects often masked possible genetic contributions, or too few genotypes were included for a robust analysis. Yang et al. (2009) looked at 31 accessions, but the plants were at different developmental stages when harvested complicating interpretation of the results. Experiments designed to specifically address genotype differences for N recycling have focused on differences between upland and lowland cultivars, the two major switchgrass cytotypes, with lowland accessions appearing to have greater N recycling (Porter, 1966; Yang et al., 2009). Recent work has shown that gene flow does occur between upland and lowland cultivars, despite differences in flowering time (3–4 weeks), which provides a mechanism for generating allelic variability (Zhang et al., 2011).

## Flowering Time and Dormancy in Switchgrass

**Figure 1:**
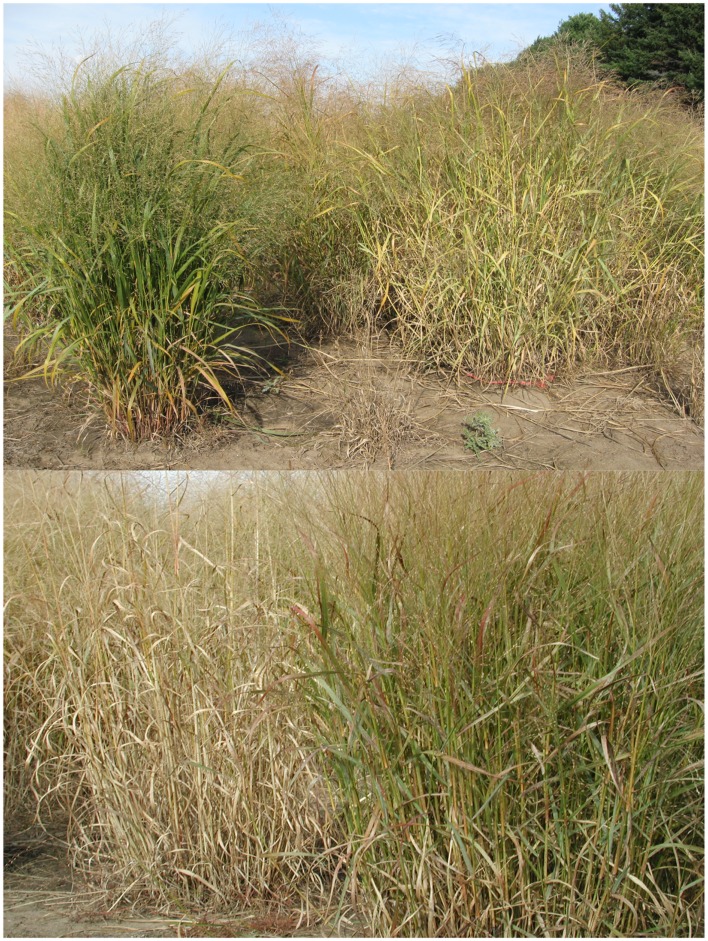
**Switchgrass varieties in a common garden with differences in senescence**.

Determining the factors that affect flowering time and dormancy in switchgrass is likely to be difficult, due to the extensive natural variation and genetic complexity within the species. Environmental factors to be considered include temperature and precipitation, which are variable from year to year, and photoperiod. Common garden experiments show that ecotypes of switchgrass are locally adapted, and the timing of reproductive development is correlated to the length of the local growing season (Cornelius and Johnston, 1941; Eberhart and Newell, 1959; McMillan, 1959, 1965; Hopkins et al., 1995; Sanderson and Wolf, 1995; Casler et al., 2004, 2007b; Berdahl et al., 2005; Casler, 2005). Variation also exists for leaf appearance rate, and end-of-season dormancy (Figure 1), all of which influences the length of active growth and biomass accumulation (McMillan, 1959; Van Esbroeck et al., 1997, 2004).

Spring emergence begins the growing season and is mostly a function of temperature (Sanderson and Wolf, 1995). McCarty (1986) showed that in native fields there was more annual variation in spring emergence than anthesis. However, there is a genetic component since the Alamo cultivar is the first to emerge in the spring in common garden experiments, and northern ecotypes are delayed compared to southern ecotypes (Hsu et al., 1985; Parrish and Fike, 2005).

Vegetative growth creates the greatest biomass accumulation and ends with initiation of floral development. Vegetative growth is also influenced by temperature, with lower temperatures increasing the duration of the vegetative stage (Benedict, 1940; Sanderson and Wolf, 1995). There is also variability among switchgrass cultivars for photoperiod sensitivity in the vegetative stage; in some cultivars flowering is inhibited by short days. For example, Alamo, a southern lowland, had twice the length of vegetative growth compared to CIR (Cave-In-Rock), an upland variety from Illinois (Sanderson et al., 1996; Van Esbroeck et al., 2003). The photoperiod effect also explains the cessation of vegetative growth observed in northern cultivars in early summer when grown in southern locations (Sanderson et al., 1996).

Benedict (1940) showed that a single ecotype of switchgrass flowered under short-day conditions (10 h), but not in long-day conditions (18 h), thus classifying switchgrass as a short-day plant. In a common garden experiment, McMillan (1959) demonstrated substantial variation for floral initiation within and between eight switchgrass populations from different locations. In a native field experiment over 15 years, anthesis was largely controlled by photoperiod, with little year to year variation (McCarty, 1986). Hopkins et al. (1995) demonstrated the strong photoperiod effect with Midwestern accession having nearly identical heading dates for 2 years at three locations with similar latitude. However, within population variability also exists since natural populations contain plants entering anthesis over a 3 week period (Jones and Brown, 1951).

Development and flowering time in switchgrass has been recorded in a number of studies, which reveal latitudinal adaptation reflected by higher survival rates among local populations in reciprocal transplant experiments (Sanderson et al., 1999; Casler et al., 2004). Thus, flowering time or maturity is highly variable among switchgrass varieties, with photoperiodic differences along a north–south gradient (McMillan, 1959; Casler et al., 2004, 2007b; Casler, 2005). There also appears to be variability in the length of the flowering period with northern clones having 1 week between inflorescence exsertion and initial anthesis, while southern clones took 4–6 weeks (McMillan, 1959). One study (Van Esbroeck et al., 2003) showed that “photoperiod did not appear to affect the initiation of reproductive development but rather the period of panicle exsertion.”

**Figure 2:**
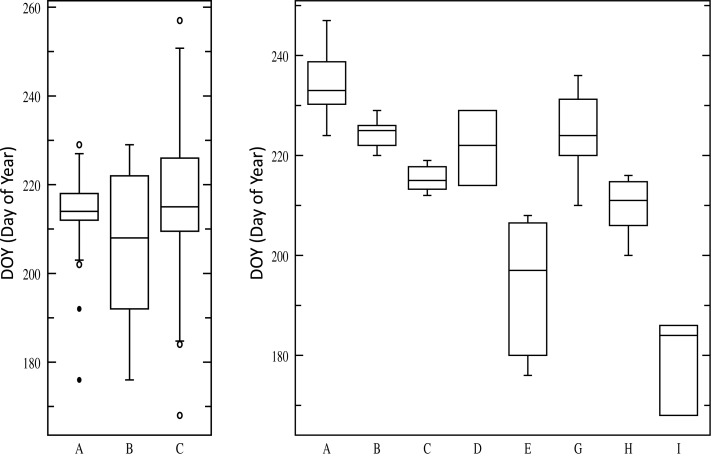
**ANOVA (Analysis of Variance) of heading date for 113 switchgrass germplasms**. Left: three groups based upon cytotype and ploidy (Lowland4×, Upland4×, Upland8×). Right: eight groups based upon cytotype, ploidy, and morphological characteristics.

The evidence from multiple studies indicates that the two major cytotypes of switchgrass, upland, and lowland, have differences in photoperiod sensitivity. For example, CIR, an upland northern cultivar, showed the largest response to an artificially extended photoperiod (18 h) in a greenhouse with an increased yield of 129 and 98% in two trials, while Alamo showed no change (Van Esbroeck et al., 2003). In general in common garden experiments, lowland ecotypes have a heading date 2–4 weeks later than upland types (Casler et al., 2004; Cortese et al., 2010). In work from Taliaferro (2002), heading date was recorded for 113 switchgrass germplasms with variable flowering time [167–257 Days Of Year (DOY) (Taliaferro, 2002)]. Grouping accessions according to cytotype and ploidy generates three significantly different groups (Lowland4×, Upland4×, Upland8×) when evaluating heading data (*P* = 0.0002), however, most variation is within each class (Figure 2). Using additional descriptive parameters for Cluster analysis, such as morphological differences, generates nine core groups, and the ANOVA in Figure 2 shows the extensive natural variability of heading dates for eight of the groups (one group, DOY248 and DOY257, is excluded due to sample size *n* = 2). On average the photoperiod effect results in 0.8 day earlier heading for each degree north a switchgrass population is moved (Casler et al., 2007b).

Flowering time has a large influence on biomass yield, with southern (lowland) varieties producing 2–3× more phytomers (root and shoot meristems) compared to northern (upland) varieties when grown in their native locations, partially related to a longer growing season (Casler, 2012). The strong adaptation gradient results in some lowland varieties never flowering at northern locations and lacking cold tolerance, and upland varieties being heat intolerant and flowering too early for maximal biomass in southern locations (Sanderson et al., 1999; Casler et al., 2004, 2007a). In fact most switchgrass varieties are well adapted to their local environment and cannot be moved more than one hardiness zone without adverse affects on productivity and survival (Casler et al., 2004). The genetic parameters that control flowering are unknown and investigation into the endogenous and exogenous factors influencing flowering time will be valuable for developing region-specific cultivars. Very few of the studies in Table 1 acknowledge flowering time differences; thus, to relate the data from different studies, samples should be collected at a uniform stage of development, and flowering time data on cultivars and locations are needed to identify the genetic components controlling N recycling.

End-of-season dormancy is especially important for N recycling. Among C4 species, McMillan (1965) noted that “early flowering switchgrass and big bluestem from the northern USA exhibited earlier dormancy than ecotypes originating in southern USA.” In photoperiod experiments with multiple accessions, 12.5 h of light affected the two earliest flowering ecotypes differently, with clones from Minnesota going dormant while clones from Colorado continuing vegetative growth. Castro et al. (2011) determined that the photoperiod at emergence was key for the timing of dormancy, and growing upland cytotypes in 24 h of light prevented dormancy. Thus there is natural variation for photoperiodic-induced dormancy with northern ecotypes having a greater response to photoperiod, likely as a mechanism to avoid freeze damage (Benedict, 1940; Van Esbroeck et al., 2004).

Photoperiod sensitivity has an influence on nearly every stage of development, and there is substantial natural variation that can be utilized for breeding and identification of the molecular components controlling photoperiod sensitivity. Together, these studies show that photoperiod sensitivity not only varies among cultivars, but also varies with developmental phase. Increasing photoperiod insensitivity could increase yield in northern ecotypes provided proper nutrient recycling can be maintained.

## Current and Future Directions

The effects of a nitrogen gradient on root architecture were evaluated in two *Brachypodium* accessions where heritable differences in root system architecture were dependent on N concentration (Ingram et al., 2012). However, evaluating growth of eight divergent *Brachypodium* accessions grown under eight different N concentrations failed to identify accession differences, and final N content was mostly influenced by flowering time, with later flowering accessions producing more leaves and thus having more total N (Schwartz and Amasino, unpublished). Since *Brachypodium* is an annual plant, both of these experiments more likely address N uptake, and not internal N recycling.

Investigating N recycling would be greatly facilitated by identifying a high-throughput and genetically amenable system to study that has robust end-of-season recycling. A perennial model system would enable the study of N recycling to the crown and roots. Hopefully such a model could undergo the yearly life cycle in a highly controlled environment (greenhouse) or in common garden experiments, thus reducing the environmental variation for identification of genetic differences. Reciprocal transplant experiments are another method to identify genetic variability. To thoroughly investigate perennialism and manipulate perennial traits, however will likely require multiple systems to investigate due to general variability for this trait (i.e., the temperate perennial life history probably arose independently multiple times).

Determining what cues initiate N recycling in perennials and how the recycling rate is controlled will be key to manipulate N recycling. Both annuals and perennials have the ability to recycle nitrogen for growth throughout the season, but temperate perennials differ by having two sinks for translocating N at the end of the season, the seeds (acropetal) and the crown/root system (basipetal). Thus some exogenous or endogenous factor promotes translocation downward in perennials in the second half of the growing season. The trigger could be the initiation of flowering, and/or changes in photoperiod, or simply robust growth in the crown and roots, which creates a sink. Determining how the crown and roots become a sink for nutrients is imperative to tailoring N recycling for specific crops and environments.

To make a substantial contribution to the bioenergy field, it will be important to identify the genetic basis of N dynamics. One study using a cross between perennial and annual rice discovered a transcription factor (Rhz3) required for rhizome growth (Hu et al., 2003). Determining the effects and manipulating expression of this gene in annual and perennial species may provide insight into the role of rhizomes in N recycling. Genetic differences might also be identified by tissue-specific expression studies. For example, a developmental profile of rhizomes over a season might help assess their role in N recycling, and how that sink is activated mid season.

Intraspecific crosses between upland and lowland cultivars show hybrid vigor (heterosis) for many traits, including biomass, indicating a rich source of genetic variation in switchgrass (Casler, 2012). Analyses of segregating populations derived from such wide crosses may be one strategy to make progress in understanding biomass traits at the molecular level.

The N dynamics of a given species in a given environment may be a critical factor in biofuel profitability. Future genetic and biochemical studies of N recycling and the control of the initiation of flowering and dormancy have great potential to increase the yield and sustainability of bioenergy crops.

## Conflict of Interest Statement

The authors declare that the research was conducted in the absence of any commercial or financial relationships that could be construed as a potential conflict of interest.
